# The ‘bystander’ stenosis dilemma: a case against revascularization in a territory of extensive myocardial scar—a case report

**DOI:** 10.1093/ehjcr/ytag153

**Published:** 2026-04-13

**Authors:** Samson S Badalyan, Syune V Markosyan

**Affiliations:** Department of Cardiovascular Surgery, GBUZ SK ‘City Clinical Hospital’, 22 Pirogova Street, Pyatigorsk 357500, Russia; Department of Cardiovascular Surgery, GBUZ SK ‘City Clinical Hospital’, 22 Pirogova Street, Pyatigorsk 357500, Russia

**Keywords:** MINOCA, Silent infarction, Cardiac magnetic resonance, Apical thrombus, Bystander stenosis, Case report

## Abstract

**Background:**

Myocardial infarction with non-obstructive coronary arteries (MINOCA) is a working diagnosis that requires careful exclusion of alternative ischaemic mechanisms. This case highlights a MINOCA mimic: a silently evolved, spontaneously reperfused transmural infarction, where cardiac magnetic resonance (CMR) was decisive for clarifying the mechanism and guiding management.

**Case summary:**

A 38-year-old man with heavy smoking as his sole cardiovascular risk factor underwent a routine evaluation. ECG demonstrated features of a large anterior infarction, while cardiac biomarkers were negative. Echocardiography showed severe left ventricular dysfunction and a large apical thrombus. Angiography revealed a partially recanalized mid-left anterior descending (LAD) and a severe diagonal stenosis without an obvious culprit lesion. CMR demonstrated a large transmural, non-viable LAD territory infarction with microvascular obstruction. A genetic panel revealed polymorphisms potentially contributing to a prothrombotic condition. Given the absence of viability, revascularization was deferred. At 6-month follow-up, the patient remained asymptomatic, with persistent apical thrombus despite anticoagulation.

**Discussion:**

Silent, spontaneously reperfused infarction may mimic MINOCA when angiography reveals only mild or intermediate lesions. CMR is essential for identifying non-viable myocardium, avoiding misclassification, and guiding management. Young patients with extensive infarction may benefit from targeted thrombophilia evaluation.

Learning points

- Spontaneously reperfused infarction can mimic MINOCA.
- Negative troponin does not exclude a large, old infarction.
- CMR is essential for determining myocardial viability.
- Do not revascularize non-viable myocardium.
- Thrombophilia testing may be useful in young patients.

## Introduction

MINOCA accounts for 5%–8% of acute coronary syndromes; however, its diagnosis requires the exclusion of obstructive coronary artery disease (CAD) and the identification of a specific ischaemic mechanism.^1,2^ A particularly challenging diagnostic scenario arises when coronary angiography reveals only non-obstructive or moderately stenotic lesions in the setting of extensive myocardial injury. Distinguishing true MINOCA from a spontaneously recanalized occlusive infarction is critical, as their underlying mechanisms, prognosis, and management strategies differ substantially.

We present an instructive case of a young, asymptomatic man with a large transmural left anterior descending (LAD) territory infarction, an apical thrombus, and angiographic findings discordant with the extent of myocardial damage (a partially recanalized LAD and a severe ‘bystander’ stenosis), ultimately explained by thrombotic occlusion followed by spontaneous reperfusion in the context of a prothrombotic genetic profile.

## Summary figure

**Figure:**
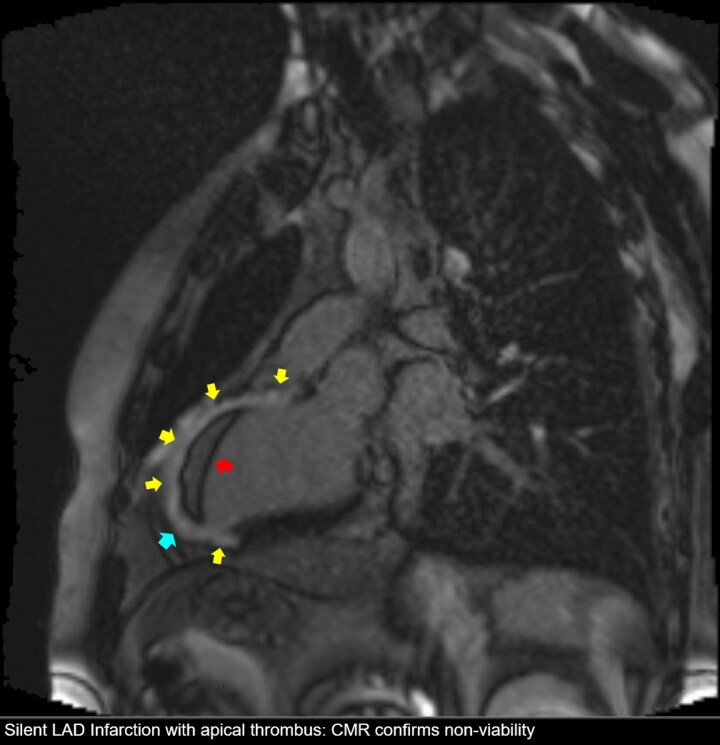


## Case presentation

A 38-year-old man, whose only cardiovascular risk factor was heavy smoking (one pack/day), presented for a routine check-up. He was entirely asymptomatic, specifically denying any history of chest pain, dyspnoea, or palpitations. The clinical evaluation was initiated solely based on an abnormal resting ECG.

**Figure 1:**
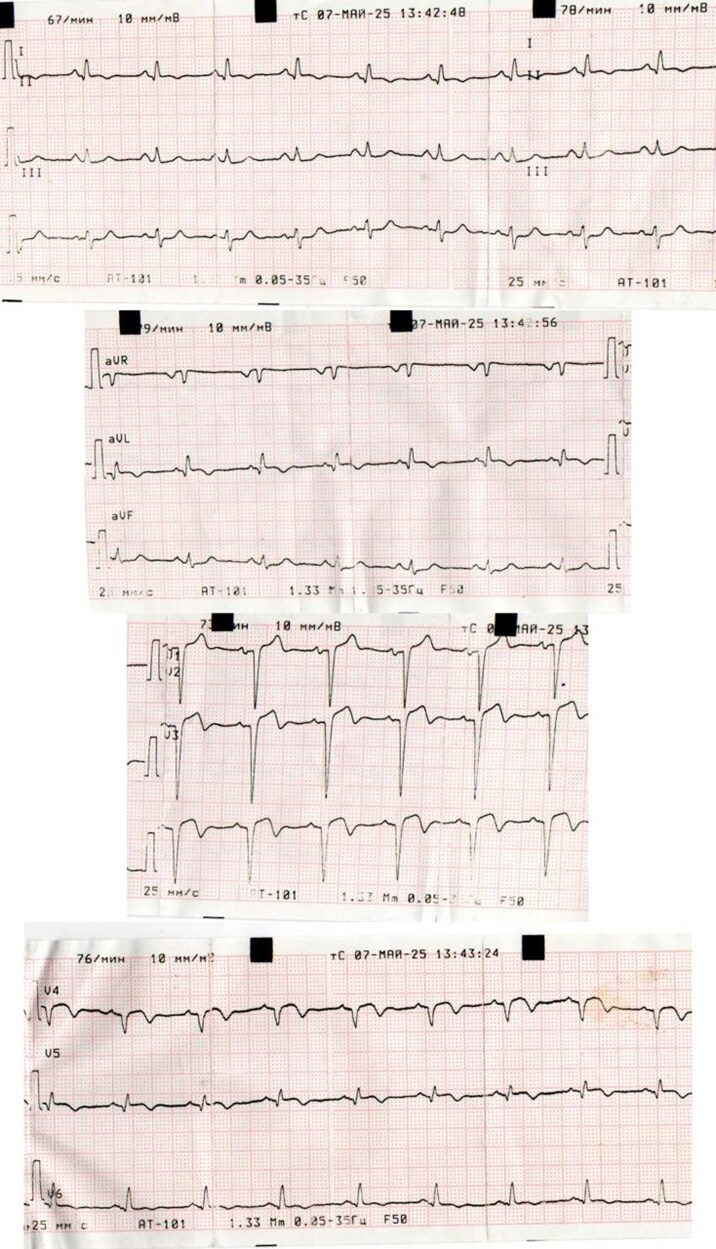
Twelve-lead electrocardiogram showing fixed T-wave inversions and pathological Q waves in the anterior leads (I, aVL, V1–V6), consistent with a prior anterior myocardial infarction. Persistent ST-segment elevation and left axis deviation indicate chronic myocardial injury and left ventricular aneurysm formation.

Physical examination revealed normal vital signs and no abnormalities on cardiac or pulmonary auscultation. A resting 12-lead ECG demonstrated deep T-wave inversions in leads I, aVL, and V2–V6, QS complexes in the anterior leads, and subtle ST-segment elevations in the anterior–lateral precordial leads (*Figure 1*). The patient's laboratory results are summarized in *Table 1*. High-sensitivity troponin I was negative, while NT-proBNP was mildly elevated at 363 pg/ml, suggesting chronic ventricular stress rather than ongoing necrosis.

**Table 1:** Summary of laboratory findings at presentation

Parameter	Patient Value	Reference Range	Comment
Cardiac biomarkers			
High-sensitivity troponin I	<5 ng/L	<15 ng/L	
NT-proBNP	363 pg/ml	<125 pg/ml	Elevated, indicating chronic heart failure
Lipid profile			
Total cholesterol:	4.2 mmol/L	<5.2 mmol/L	Optimal
LDL-C	2.2 mmol/L	<3.0 mmol/L	Optimal
HDL-C	1.4 mmol/L	>1.0 mmol/L	Optimal
Triglycerides	1.1 mmol/L	<1.7 mmol/L	Optimal
Lipoprotein (a)	0.2 g/L	<0.5 g/L	Normal
Renal function and glucose			
Creatinine	85 μmol/L	60–110 μmol/L	
eGFR	>90 ml/min/1.73m^2^	>90 ml/min/1.73 m^2^	
Blood glucose	4.3 mmol/L	3.9–5.5 mmol/L	
Glycated haemoglobin (HbA1c)	4.9%	<6.0%	
Plasma homocysteine	13.1 μmol/L	<15 μmol/L	
Genetic thrombophilia screen			
Antithrombin III	105%	80%–120%	Normal
Protein C activity	98%	70%–130%	Normal
Protein S activity	112%	65%–140%	Normal
Lupus anticoagulant	Negative	Negative	
Anticardiolipin antibodies	Negative	<10 U/ml	
Factor V Leiden mutation	Not detected	Wild type	
Prothrombin G20210A mutation	Not detected	Wild type	
Genetic thrombophilia panel			
PAI—1 4G/G genotype	Detected	–	
MTHFR 677C/T	Detected	–	
FGB-455 G/A	Detected	–	

**Figure 2:**
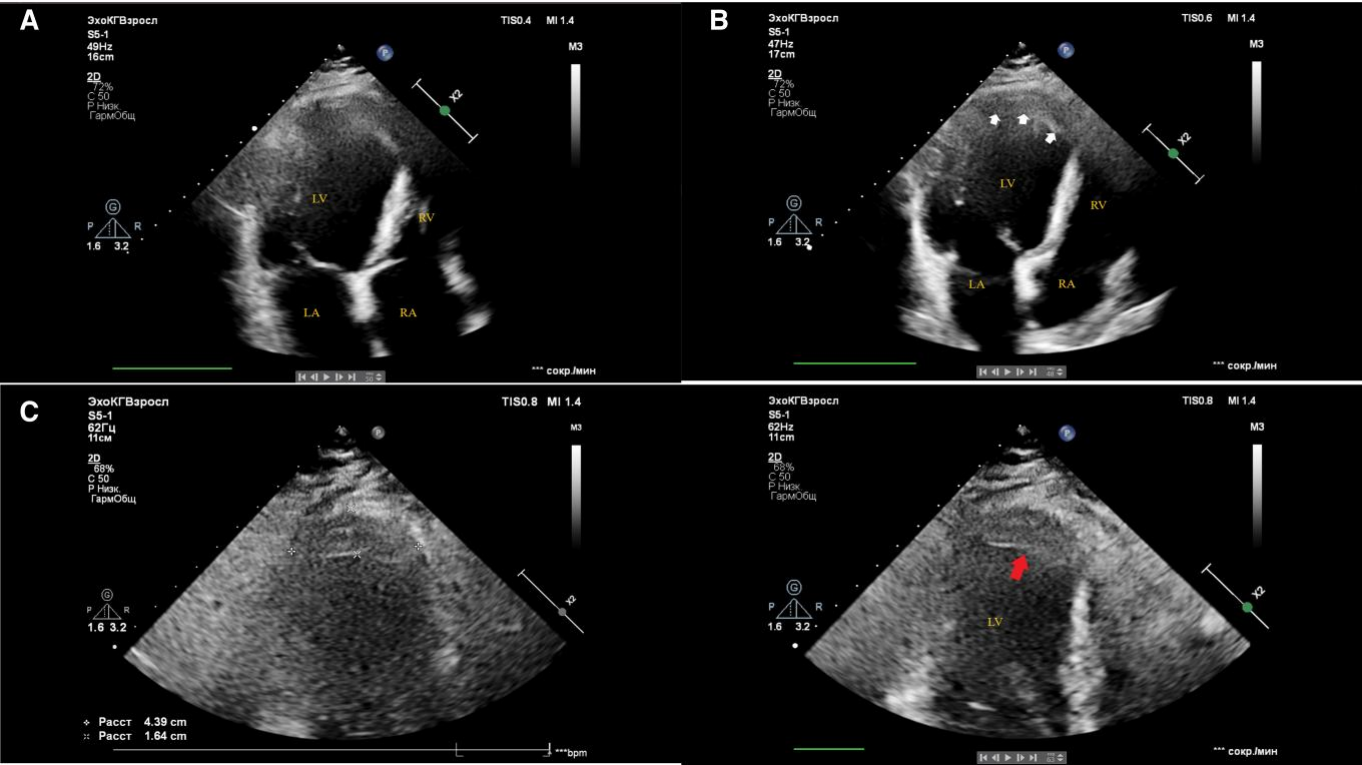
Transthoracic echocardiography findings. *(A)* Apical four-chamber view showing overall cardiac anatomy. *(B)* Apical four-chamber view demonstrating a parietal apical thrombus (white arrow) and apical-septal aneurysm. *(C)* Apical two-chamber view showing the dimensions of the apical thrombus (dotted crosses). *(D)* Apical two-chamber view highlighting the apical thrombus (red arrow) and left ventricular apical-septal aneurysm. These images demonstrate marked left ventricular dilation, apical akinesia, reduced contractility, and a left ventricular ejection fraction of 35%, consistent with severe systolic dysfunction.

Transthoracic echocardiography revealed severe left ventricular systolic dysfunction with an ejection fraction of 35%, an end-diastolic volume of 210 ml, and an end-systolic volume of 137 ml (*Figure 2*). There was extensive akinesia of the anterior and septal walls, and a large mural thrombus was identified at the apex. Carotid duplex imaging showed no significant stenosis.

**Figure 3:**
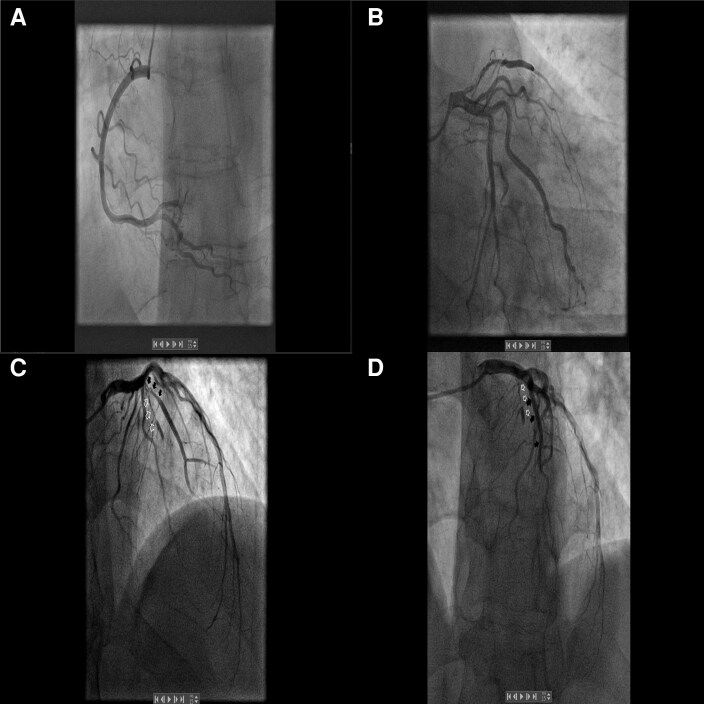
Coronary angiography. *(A)* Right coronary artery injection showing a normal, right-dominant coronary circulation. *(B)* Left circumflex artery angiogram without significant lesions. *(C)* Left anterior descending (LAD) artery with a partially recanalized thrombus (‘ghost sign’, white arrow). *(D)* Large diagonal branch with a critical 90% ostial stenosis (black arrow). The LAD supplies a territory of transmural scar, confirming the futility of revascularization in this region.

Given these findings, the patient was hospitalized and started on guideline-directed medical therapy, which included bisoprolol, sacubitril/valsartan, dapagliflozin, eplerenone, aspirin, and atorvastatin, alongside anticoagulation with warfarin (target INR 2–3). Warfarin was chosen as it remains the standard of care for left ventricular thrombus in our institution, in line with traditional guidelines and local practice. Although the patient’s LDL cholesterol level was within the normal range, statin therapy was initiated in accordance with current ESC guidelines for secondary prevention after myocardial infarction. This approach is supported by the pleiotropic, anti-inflammatory, and plaque-stabilizing effects of statins, which are independent of baseline LDL levels. Coronary angiography revealed a partially recanalized mid-LAD segment and a severe (90%) stenosis of a diagonal branch (*Figure 3*). No other obstructive lesions were identified, and there was no clear culprit lesion that could account for the extensive myocardial injury.

**Figure 4:**
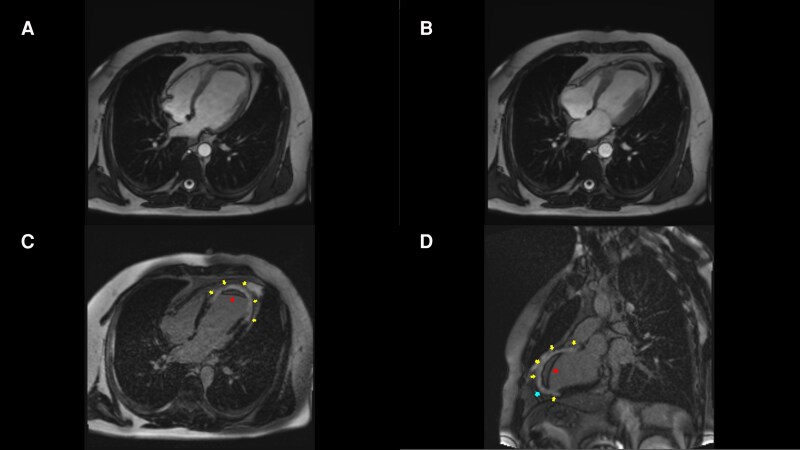
Cardiac magnetic resonance imaging (LGE). *(A)* End-diastolic and *(B)* end-systolic views illustrating global remodelling of the left ventricle. *(C)* Late gadolinium enhancement (LGE) image showing transmural scarring of the anterior wall (yellow arrow) and an apical thrombus (red arrow). *(D)* LGE sequence demonstrating left ventricular aneurysm (blue line) and non-viable myocardium. Findings confirm extensive transmural fibrosis and the presence of a left ventricular apical thrombus.

Subsequent cardiac magnetic resonance (CMR) imaging confirmed a large transmural infarction in the LAD and diagonal territories, with associated microvascular obstruction and a persistent apical thrombus (*Figure 4*). A genetic thrombophilia panel identified PAI-1 4G/4G, MTHFR 677C/T, and FGB −455 G/A polymorphisms, suggesting a prothrombotic predisposition that likely contributed to the thrombotic event. Given the absence of viable myocardium in the affected territories, revascularization of either the LAD or the diagonal branch was not performed.

A formal cardio-surgical consultation concluded that neither surgical aneurysm resection nor coronary artery bypass grafting/percutaneous coronary intervention was indicated, as these procedures would offer no functional or prognostic benefit in the setting of transmural non-viability and would pose an unacceptable risk. Anticoagulation was initiated with warfarin. However, after approximately 1 month of therapeutic anticoagulation without evidence of thrombus resolution on follow-up echocardiography, the therapy was switched to rivaroxaban 20 mg daily, based on recent reports of DOAC efficacy in this setting, acknowledging the off-label nature of this approach.

At the 6-month follow-up, the patient remained asymptomatic and fully adherent to guideline-directed medical therapy, including aspirin, bisoprolol, dapagliflozin, eplerenone, sacubitril/valsartan, atorvastatin, and rivaroxaban. The patient had successfully quit smoking. Follow-up echocardiography was performed at 6 months and confirmed persistent severe left ventricular dysfunction with no significant change. The apical thrombus persisted despite ongoing therapeutic anticoagulation with rivaroxaban.

## Discussion

This case underscores several clinically pivotal concepts regarding silent myocardial infarction, the interpretation of biomarkers, the MINOCA paradigm, and viability-guided management.

### The diagnostic challenge: silent infarction with negative biomarkers

The patient’s presentation constituted a diagnostic paradox: electrocardiographic evidence of a massive anterior infarction juxtaposed with negative troponin and a complete absence of symptoms. This highlights a critical clinical caveat: troponin is a marker of acute myonecrosis, not of historical scar formation.^1,3^ In delayed presentations, normalized biomarkers shift the diagnostic burden to electrocardiography and advanced imaging, which were pivotal in revealing the true extent of this silent, completed infarction.

### Reclassifying MINOCA: the pivotal role of cardiac MRI

While the initial finding of non-obstructive coronary arteries on angiography fittingly suggested MINOCA,^1,4,5^ gadolinium-enhanced CMR served as the ultimate arbiter, enabling a definitive reclassification. The presence of a large, transmural scar with microvascular obstruction is pathognomonic of an acute occlusive infarction followed by spontaneous reperfusion—a finding incompatible with classic MINOCA aetiologies such as vasospasm or myocarditis. This case emphasizes that CMR is not merely an adjunct but an essential tool for delineating the underlying pathophysiology in cases of suspected MINOCA.^6–8^

### The etiological Enigma: contribution of a prothrombotic state

In a young patient whose only traditional risk factor was smoking, the search for an aetiology logically extended to thrombophilia testing. The identified combination of polymorphisms (PAI-1 4G/4G, MTHFR 677C/T, FGB-455 G/A) is not typically classified as a classic inherited thrombophilia. However, it may collectively create a prothrombotic milieu by impairing fibrinolysis and increasing fibrinogen levels. In this context, this genetic background represents a plausible significant predisposing factor that likely interacted with smoking—a potent endothelial toxin—to precipitate the occlusive coronary event. It is important to note that the clinical significance of these polymorphisms in coronary thrombosis remains a subject of ongoing research, and their identification in this case should be interpreted as a correlation rather than a definitive causation.^9^

### The management dilemma: bystander stenosis in non-viable myocardium

A central management tenet demonstrated here is the futility of revascularizing stenoses that supply non-viable myocardium. Despite the presence of a 90% stenosis in the diagonal branch, CMR confirmed complete transmural non-viability of the entire LAD territory. Thus, this severe stenosis represented a ‘bystander lesion’. Revascularization would therefore offer no functional or prognostic benefit while still exposing the patient to procedural risks. This decision is firmly supported by viability-guided management principles endorsed in major guidelines^1,2^ and corroborated by trial data.^10,11^

### The clinical challenge of a persistent apical thrombus

The formation and persistence of the apical thrombus, despite therapeutic anticoagulation, is a recognized consequence of a large, aneurysmal, and akinetic apical segment where blood stasis prevails. This suggests the development of an organized fibrin clot that is resistant to lysis. This management challenge shifts the therapeutic focus towards long-term anticoagulation for embolic prophylaxis and strict rhythm control to minimize stasis, rather than expecting complete resolution of the thrombus.

### Integrated clinical lessons

This case provides a diagnostic paradigm for approaching MINOCA mimics, demonstrating how multimodal imaging—especially CMR—coupled with a thorough investigation for prothrombotic states can prevent misclassification and guide appropriate management, thereby averting unnecessary and potentially harmful interventions.

ICD therapy was discussed with the patient but deferred given the absence of documented ventricular arrhythmias and stable clinical status on optimal medical therapy, in accordance with current guidelines.

## Study limitations

This report shares the inherent limitation of a single-case observation, which precludes broad generalizations. The clinical significance of the identified genetic polymorphisms should be interpreted as correlative rather than definitively causative. Although a formal haematology consultation was considered, the identified polymorphisms are not associated with a clear thrombotic risk in current guidelines; therefore, the patient was managed by cardiology, with the genetic findings interpreted as a possible contributing factor rather than a definitive aetiology. Furthermore, the follow-up period was limited to 6 months; long-term monitoring is ongoing.

## Conclusion

This case illustrates a rare presentation of a large, silent, transmural LAD infarction mimicking MINOCA in a young man with a prothrombotic genetic profile. CMR was decisive in identifying the true mechanism of injury,^6–8^ differentiating a recanalized thrombotic occlusion from non-ischaemic or microvascular causes. Viability-guided management appropriately prevented futile revascularization of the non-viable myocardium.^10,11^ This report reinforces the critical importance of integrating advanced cardiac imaging and thrombophilia assessment in cases where angiographic findings appear discordant with the extent of myocardial damage.

## Patient perspective

‘I felt completely healthy and did not expect anything serious when I came for a routine ECG. I am grateful that the problem was discovered, allowing treatment to begin promptly. The clear explanations and consistent follow-up from the medical team have helped me understand the importance of quitting smoking and taking my medications regularly’.

## Lead author biography


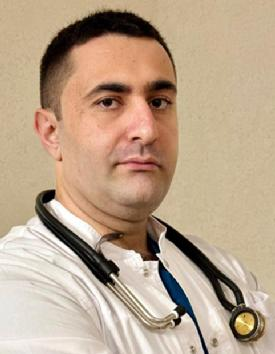


Samson S. Badalyan, MD, PhD, is a cardiologist at the City Clinical Hospital in Pyatigorsk, Russia. His clinical practice involves the diagnosis, treatment, and long-term management of patients with a wide range of cardiovascular conditions, including coronary artery disease, heart failure, arrhythmias, and structural heart disease, as well as the perioperative care of cardiac surgery patients. He has extensive experience in non-invasive imaging (echocardiography, transoesophageal echocardiography including intraoperative monitoring) and ECG interpretation.

## Data Availability

The data underlying this article are available in the article and in its online Supplementary material.

## Notes

**Consent:** Written informed consent was obtained from the patient for the publication of this case report and any accompanying images.
